# Discovery and Validation of a Three-Cytokine Plasma Signature as a Biomarker for Diagnosis of Pediatric Tuberculosis

**DOI:** 10.3389/fimmu.2021.653898

**Published:** 2021-04-16

**Authors:** Nathella Pavan Kumar, Syed Hissar, Kannan Thiruvengadam, Velayuthum V. Banurekha, N. Suresh, Janani Shankar, Elilarasi S, Gomathi N S, Kalpana S, Ganesh J, Aravind M A, Dhanaraj Baskaran, Srikanth Tripathy, Soumya Swaminathan, Subash Babu

**Affiliations:** ^1^ National Institutes of Health, National Institute for Research in Tuberculosis, International Center for Excellence in Research, Chennai, India; ^2^ Indian Council of Medical Research, National Institute for Research in Tuberculosis, Chennai, India; ^3^ Department of Pediatrics, Kanchi Kamakoti CHILDS Trust Hospital, Chennai, India; ^4^ Department of Pediatrics, Institute of Child Health and Hospital for Children, Chennai, India; ^5^ Department of Pediatrics, Government Stanley Medical College and Hospital, Chennai, India; ^6^ Public Health Division, World Health Organization, Geneva, Switzerland; ^7^ Laboratory of Parasitic Diseases, National Institute of Allergy and Infectious Diseases, National Institute of Health, Bethesda, MD, United States

**Keywords:** pediatric tuberculosis, cytokines, biomarkers, ELISA, tuberculosis diagnostics, anti-tuberculosis treatment

## Abstract

Pediatric TB poses challenge in diagnosis due to the paucibacillary nature of the disease. We conducted a prospective diagnostic study to identify immune biomarkers of pediatric TB and controls (discovery cohort) and obtained a separate “validation” cohort of confirmed cases of pediatric TB and controls. Multiplex ELISA was performed to examine the plasma levels of cytokines. Discovery and validation cohorts revealed that baseline plasma levels of IFNγ, TNFα, IL-2, and IL-17A were significantly higher in active TB (confirmed TB and unconfirmed TB) in comparison to unlikely TB children. Receiver operating characteristics (ROC) curve analysis revealed that IFNγ, IL-2, TNFα, and IL-17A (in the discovery cohort) and TNFα and IL-17A (in the validation cohort) could act as biomarkers distinguishing confirmed or unconfirmed TB from unlikely TB with the sensitivity and specificity of more than 90%. In the discovery cohort, cytokines levels were significantly diminished following anti-tuberculosis treatment. In both the cohorts, combiROC models offered 100% sensitivity and 98% to 100% specificity for a three-cytokine signature of TNFα, IL-2, and IL-17A, which can distinguish confirmed or unconfirmed TB children from unlikely TB. Thus, a baseline cytokine signature of TNFα, IL-2, and IL-17A could serve as an accurate biomarker for the diagnosis of pediatric tuberculosis.

## Introduction

Tuberculosis (TB) is responsible for the one of the highest number of deaths from a single infectious disease worldwide (1). Pediatric TB is estimated to constitute approximately 10% to 20% of the total TB burden in high burden countries (2, 3). However, the reported global burden of TB in children remains an underestimate. This is mainly because of the greater challenge in confirming the diagnosis of pediatric TB due to the paucibacillary nature of TB disease in children (4). Children who are under the age of 5 years are thought to present comparatively weaker immune responses against tuberculosis infection compared to adults (5), which in turn leads to increased susceptibility to *Mycobacterium tuberculosis* (*M.tb*) infection and disease (6). In contrast, adolescents present with exaggerated immune responses leading to increased pathology and more severe disease (7). Previous studies on TB diagnosis are executed largely in adult populations and then applied to children. Since both groups have distinct immune and inflammatory profiles, the established tests are not as accurate for diagnosis of pediatric TB (8). The significance of successful diagnostics in young children cannot be overstated, as *M.tb* infection is difficult to identify and tends to progress quickly and often to severe TB disease, if left untreated (9).

Diagnosis of pediatric TB remains challenging with the current routine clinical and laboratory diagnostic tools. There is also a lack of reliable TB diagnostic tests to be used in pediatric populations in TB endemic countries. In adult studies, the sensitivity and specificity of *Xpert MTB/RIF* (Cepheid, USA) is equivalent to that of liquid culture (10–12), whereas the data from pediatric population suggest that the sensitivity of *Xpert MTB/RIF* is lower in children (13, 14). The diagnostic challenge is two-fold – 1) the pauci-bacillary nature of the disease and 2) the inability to young children to expectorate on request. The possibility of an accurate blood based assay could address both these shortcomings.

Immunological biomarker assays can bring light to this problem, but the majority of published studies investigated TB immune biomarkers in the adult population. We and others have reported on the diagnostic potential of Type 1 cytokine biomarkers to distinguish children or adults with TB infection (i.e. latent TB or active TB) from those who are uninfected (15, 16). Therefore, in this study, we aimed to determine the plasma concentrations of type 1 (IFNγ, IL-2, and TNFα), type 17 (IL-17A), and other pro-inflammatory (IL-6, GM-CSF IL-1α, and IL-1β) cytokines as markers capable of distinguishing among children who were microbiology positive (confirmed TB) or those negative (unconfirmed TB) compared with unlikely TB children in a prospectively recruited discovery cohort and subsequently blindly validated by the validation cohort.

## Materials and Methods

### Ethics Approval

All individuals were examined as part of two clinical research protocols (NIRTIEC122010 and NIRTIEC2012004) approved by Internal Ethical Committee of the ICMR-National Institute for Research in Tuberculosis, Chennai. For the discovery cohort, children suspected of TB were recruited from the Institute of Child Health and Hospital for Children, and Government Stanley Medical College and Hospital, Chennai between February 2016 to March 2018 and for the validation cohort, samples from a prior study on childhood tuberculosis (2010–2013) conducted at the Kanchi Kamakoti CHILDS Trust Hospital, Chennai were used. For the discovery cohort, ethical clearance was also obtained from Institute of Child Health and Hospital for Children, Chennai and for the validation cohort, ethical clearance was obtained from Kanchi Kamakoti CHILDS Trust Hospital, Chennai.

## Study Population and Procedures

### Discovery Cohort

Of the 195 children screened, 167 children were recruited between February 2016 to March 2018 which includes 44 children who were microbiology positive (confirmed TB) for *M.tb*, 47 children who were microbiology negative (unconfirmed TB) but were empirically treated with ATT, 76 children with other respiratory ailments with tuberculin skin (TST) positive or negative as unlikely TB controls (17). All children in the discovery cohort underwent sputum smear and culture (or *Xpert MTB/RIF*) and Tuberculin Skin Test (TST). Children with confirmed pulmonary TB were diagnosed by being positive for *Xpert MTB/RIF* or smear or solid culture for *M.tb*, with drug susceptible TB. Children with unconfirmed TB were diagnosed based on abnormal chest X-ray, history of household TB contact, positive TST and responding to anti-tuberculosis treatment (ATT). At the time of enrolment, all active TB cases (Confirmed and Unconfirmed TB together) had no record of prior TB disease or ATT. Tuberculin skin test (TST) with 2TU PPD was administered to the children. An induration of ≥10 mm in HIV-uninfected or severely malnourished with Z scores ≤ 3 SD (severely stunted/under-weight/wasted) was considered positive. Unlikely TB children who had a differential diagnosis apart from TB and who were either negative or positive for TST results were considered as controls. Unlikely TB Children who had a differential diagnosis apart from TB and who were either negative or positive for TST results were considered as controls. Unlikely TB children were children who were suspected of having TB but clinically diagnosed as having other respiratory illness like COPD, viral pneumonia, bacterial pneumonia or Asthma/wheeze empirically treated with antibiotics/antivirals/anti asthmatic medication and followed up for a period of 8 weeks for resolutions of symptoms, resolution of chest x-ray abnormalities and confirmed as TB culture negative. Information on the alternative diagnoses for each child is provided in **Supplementary Table 1**. All children were recruited from the same hospital. Most of the enrolled children were BCG vaccinated. Plasma samples were collected from all of the above children. All children were HIV negative. All confirmed and unconfirmed TB children were administered standard ATT for 6 months. At 6 months following ATT initiation, fresh plasma samples were obtained from a subset of confirmed TB (n=24) and unconfirmed TB (n=23) children (convenient sampling). All children with active TB were culture negative and symptom free at the end of ATT.

### Validation Cohort

Of the 102 children screened, a group of 36 children with confirmed TB—as well as 46 children with unlikely TB were recruited between 2010 and 2013. All children in the validation cohort underwent sputum smear and culture and Quantiferon TB Gold in Tube Assay. The diagnosis of active tuberculosis was made on the basis of being positive for sputum microscopy and solid culture with drug susceptible TB, and the samples were collected before the commencement of anti-TB treatment. Unlikely TB children were children who were suspected of having TB but clinically diagnosed as having other respiratory illness, treated empirically with antibiotics/antivirals and followed up till the complete cure of respiratory illness and receipt of negative culture reports for *M.tb*. Most of the enrolled children were BCG vaccinated. All the children were HIV negative. All active TB children received standard ATT for six months.

### Multiplex Cytokine Assay

Plasma samples from both cohorts were purified and stored frozen at −80°C prior to being subjected to Luminex assays (Bio-Rad, Hercules, CA) at the same time. Plasma levels of IFNγ, TNFα, IL-2, IL-17A, IL-1α, IL-1β, GM-CSF, and IL-6 were measured using Luminex Magpix multiplex cytokine assay system (Bio-Rad, Hercules, CA, USA). Luminex Human Cytokines Magnetic Assay kit (R & D systems, USA) was used to measure the cytokine levels. Cytokines IFNγ (lower detection limit (LDL), 51.4 pg/ml and upper detection limit (UDL), 12490 pg/ml), TNFα, (LDL, 10.1 pg/ml and UDL, 2470 pg/ml), IL-2 (LDL, 29.6 pg/ml and UDL, 7200 pg/ml), IL-17A (LDL, 12.8 pg/ml and UDL, 3110 pg/ml), IL-1α (LDL, 5.2 pg/ml and UDL, 1270 pg/ml), IL-1β (LDL, 20.2 pg/ml and UDL, 4920 pg/ml), GM-CSF (LDL, 12.2 pg/ml and UDL, 2970 pg/ml), and IL-6 (LDL, 5 pg/ml and UDL, 1220 pg/ml)

### Statistical Analysis

Geometric means (GM) were used for measurements of central tendency. Statistically significant differences between confirmed TB, unconfirmed TB and unlikely TB children were analyzed using the Kruskal-Wallis test with Dunn’s multiple comparisons. Wilcoxon signed rank test was used to compare cytokine concentrations before and after ATT. Receiver Operator Characteristics (ROC) curves was designed to test the power of each candidate cytokine immune biomarker to distinguish active TB from unlikely TB. The cut offs for the ROC curves in the two cohorts were the same. Analyses were performed using Graph-Pad PRISM Version 8.0 (GraphPad Software, CA, USA). We performed a combinatorial analysis of multiple immune biomarkers to define ideal marker combinations of the tested circulating plasma cytokines using the CombiROC method. We created all possible cytokine combinations for each confirmed and unconfirmed TB group with respect to controls in the both discovery and validation cohort. The combinations that delivered the highest sensitivity and specificity values were considered for selection of efficient immune biomarker signatures. Computation and selection of optimal biomarker combinations by integrative ROC were analyzed using freely available web application (http://CombiROC.eu) CombiROC v.1.2 (18).

## Results

### Study Population

**Figure 1:**
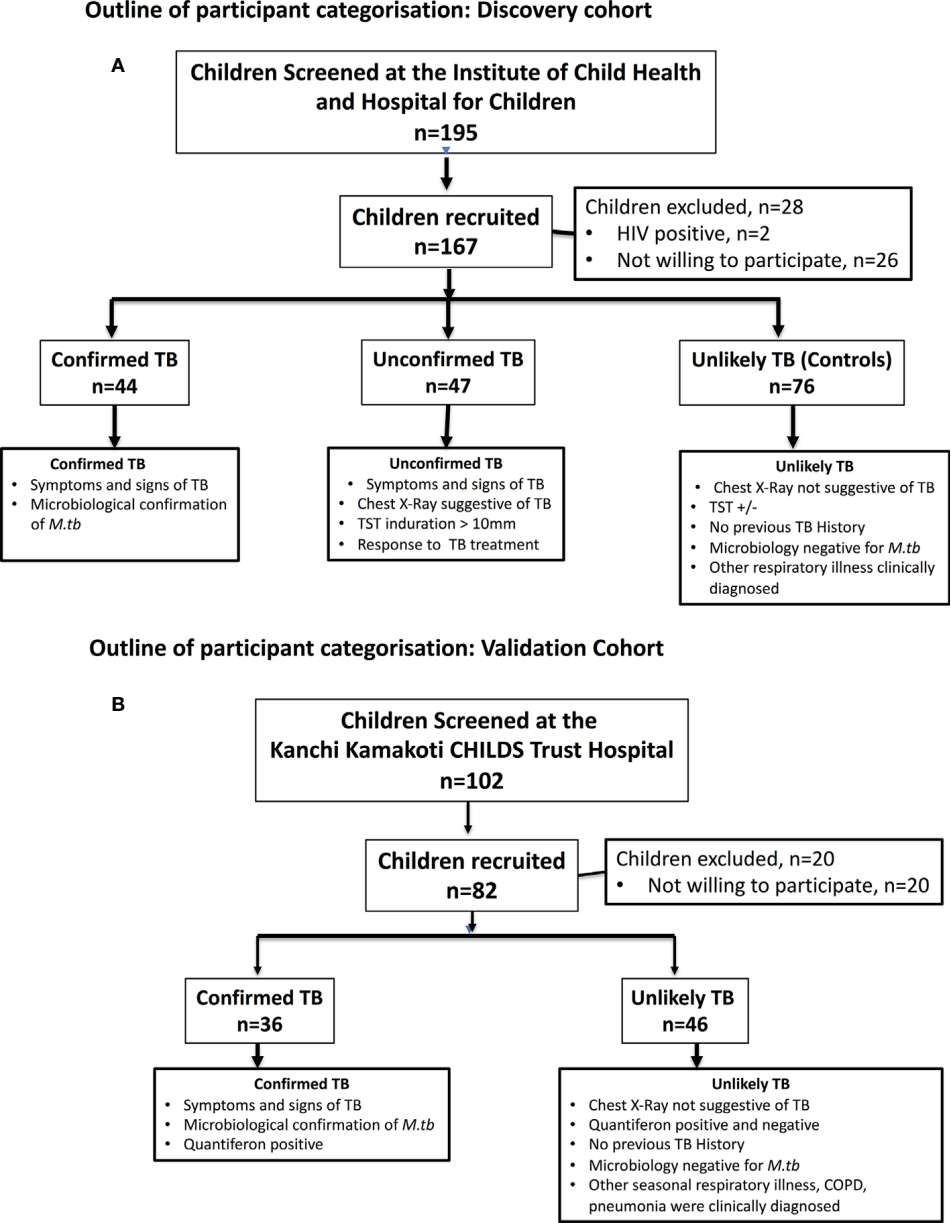
Outline of participant categorization. **(A)** Discovery cohort: In the discovery cohort (*N* = 167), plasma samples were collected from children who were microbiology positive (confirmed TB) or those negative (unconfirmed TB) for *M.tb* but with symptoms that suggested tuberculosis were studied at baseline and at the end of ATT. Children with other respiratory ailments as unlikely TB were also studied at baseline as controls. **(B)** Validation cohort: In the validation cohort (*N* = 82), plasma samples were collected from children who were microbiology positive for *M.tb* with symptoms that suggested tuberculosis were classified as confirmed TB and children with other respiratory ailments with Quantiferon negative as unlikely TB controls.

The recruitment algorithm for the children in the discovery cohort is shown in **Figure 1A**. Of the 195 children screened, 167 were recruited. The demographics of the children in the discovery cohort are shown in **Table 1**. We had 20 children below 5 years, 8 children between 5 and 10 and 16 children between 10 and 13 years in the confirmed TB group and 21 children below 5 years, 6 children between 5 and 10 years and 20 children between 10 and 12 years in the unconfirmed TB group. All children in the confirmed and unconfirmed TB group were TST positive. The recruitment algorithm for the children in the validation cohort is shown in **Figure 1B**. Of the 102 children screened, 82 were recruited. The demographics of the children in the validation cohort are shown in **Table 1**. We had 14 children below 5 years, 8 children between 5 and 10 years and 14 children between 10 and 15 years in the confirmed TB group. All confirmed TB children were Quantiferon positive.

**Table 1:** Study Demographics.

Discovery cohort			
Demographic characteristic	Confirmed TB	Unconfirmed TB	Unlikely TB
Number of subjects recruited	44	47	76
Gender (Male/Female)	28/16	28/19	36/40
Median Age (Range) (in years)	7 (1–13)	8 (1–12)	6 (1–14)
Bacterial Burden:	11/33/0	0/0/47	–
High burden/Low burden/No burden			
Tuberculin Skin Test: (Positive/Negative)	44/0	47/0	45/31
BCG Scar (Yes/No)	42/2	45/2	75/1
**Validation cohort**			
**Demographic characteristic**	**Active TB**	**Unlikely TB**	
Number of subjects recruited	36	46	
Gender (Male/Female)	19/17	28/18	
Median (range) age (in years)	9 (1–15)	9 (2–15)	
Quantiferon Assay (+/-)	36/0	10/36	
BCG Scar (Yes/No)	35/1	45/1		

Bacterial Burden Classification: at 200× focus 1+ = 3–24 AFB in one field; 2+ = 25–250 AFB in one field and 3+ = >250 AFB in one field.

### Plasma Levels of Type 1 and Type 17 Cytokines Are Elevated in Children With Active TB Disease in the Discovery Cohort

**Figure 2:**
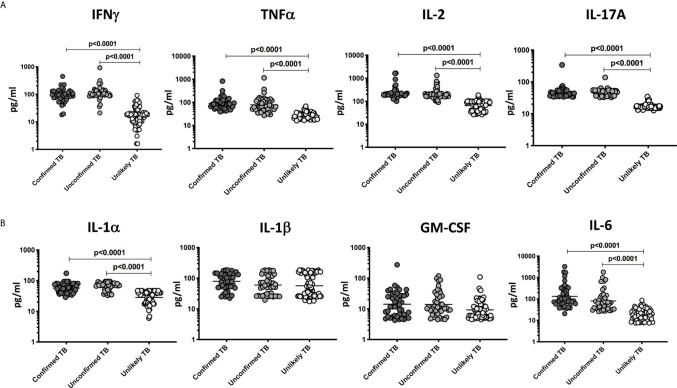
Elevated circulating levels of Type 1, Type 17 and other pro-inflammatory cytokines in children with confirmed and unconfirmed TB disease – Discovery cohort. **(A)** The plasma levels of IFNγ, TNFα, IL-2, and IL-17A and **(B)** IL-1α, IL-1β, GM-CSF, and IL-6 were measured in confirmed TB (n=44), unconfirmed TB (n=47) and unlikely TB (n=76) individuals at baseline. The data are represented as scatter plots with each circle representing a single individual. P values were calculated using the Kruskal-Wallis test with Dunn’s post-hoc for multiple comparisons.

To compare the levels of pro-inflammatory cytokines in children with active TB disease and with no TB disease (but with other respiratory illness), we measured the plasma levels of IFNγ, TNFα, IL-2, IL-17A, IL-1α, IL-1β, GM-CSF, and IL-6 in children with confirmed TB (n=44), unconfirmed TB (n=47) and unlikely TB (n=76) (**Figure 2**). As shown in **Figure 2**, the plasma levels of Type 1 and Type 17 cytokines - IFNγ, TNFα, IL-2, and IL-17A were significantly higher in children with confirmed and unconfirmed TB compared to unlikely TB children. Similarly, as shown in **Figure 2**, the plasma levels of IL-1 and IL-6 were significantly higher in children with confirmed and unconfirmed TB compared to unlikely TB children.

### Plasma Cytokines Can Robustly Distinguish Confirmed and Unconfirmed TB From Unlikely TB in the Discovery Cohort

**Figure 3:**
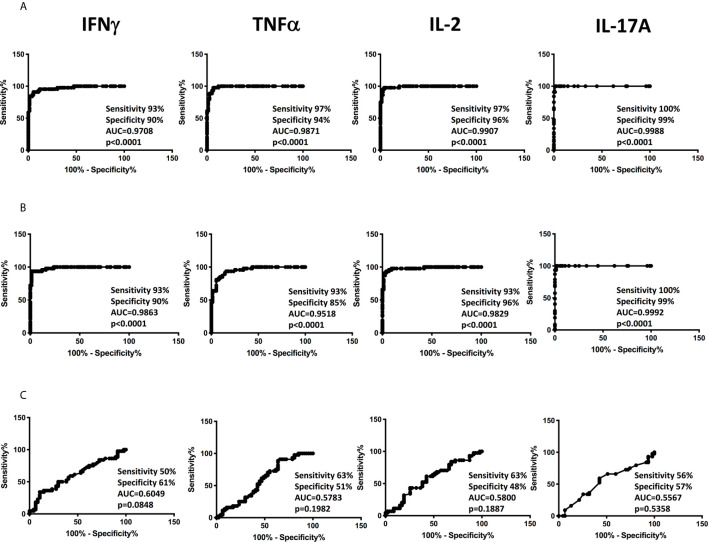
ROC analysis to estimate the discriminatory power of plasma Type 1 and Type 17 cytokines in children with confirmed or unconfirmed TB and unlikely TB – Discovery cohort. ROC analysis to estimate the sensitivity, specificity and AUC was performed using IFNγ, TNFα, IL-2, and IL-17A to estimate the capacity of these factors to distinguish individuals with **(A)** Confirmed TB vs. unlikely TB **(B)** unconfirmed TB vs. unlikely TB **(C)** Confirmed vs. unconfirmed TB.

To determine the discriminatory power of plasma Type 1 and Type 17 cytokines in distinguishing children with confirmed and unconfirmed TB from unlikely TB, we performed ROC analysis of IFNγ, TNFα, IL-2, and IL-17A in confirmed and unconfirmed TB vs. unlikely TB children and confirmed vs unconfirmed TB (**Figure 3**). As shown in **Figure 3A**, IFNγ, TNFα, IL-2, and IL-17A exhibited significant discriminatory power with high area under the curve (AUC) values, sensitivity and specificity in discriminating confirmed TB from unlikely TB children. Similarly, as shown in **Figure 3B**, IFNγ, TNFα, IL-2, and IL-17A exhibited significant discriminatory power with high AUC values, sensitivity and specificity in discriminating unconfirmed TB from unlikely TB children. Even following stratification by age, we continued to observe significant differences between confirmed/unconfirmed and unlikely TB children (**Supplementary Tables 2A, B**). Finally, as shown in **Figure 3C**, IFNγ, TNFα , IL-2, and IL-17A exhibited no significant discriminatory power between the confirmed TB vs unconfirmed TB children. Thus, plasma cytokines exhibit the potential to serve as biomarkers to distinguish both confirmed and unconfirmed TB disease from unlikely TB children.

### Plasma Levels of IFNγ, TNFα, IL-2, and IL-17A Are Elevated in Children With Confirmed TB Disease in the Validation Cohort

**Figure 4:**
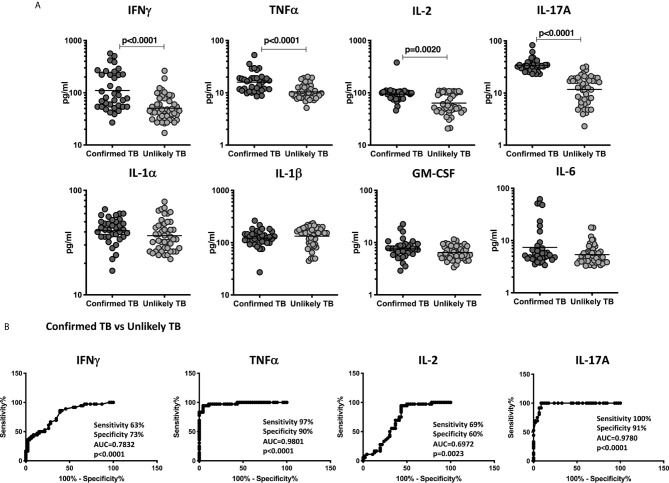
Elevated circulating levels of Type1 and Type 17 cytokines in children with confirmed TB disease and ROC analysis to estimate the discriminatory power of plasma Type 1 and Type 17 cytokines in children with confirmed TB disease and unlikely TB – Validation cohort. **(A)** The plasma levels of IFNγ, TNFα, IL-2, and IL-17A **(B)** IL-1α, IL-1β, GM-CSF, and IL-6 and were measured in confirmed TB (n=36) and unlikely TB (n=46) individuals at baseline. **(B)** ROC analysis to estimate the sensitivity, specificity and AUC was performed using IFNγ, TNFα, IL-2, and IL-17A to estimate the capacity of these factors to distinguish individuals with confirmed TB vs. unlikely TB. The data are represented as scatter plots with each circle representing a single individual. P values were calculated using the Mann-Whitney test with Holm’s correction for multiple comparisons.

To examine the diagnostic performance of the cytokine biomarkers, we applied it to blinded plasma samples from the validation cohort. Plasma levels of IFNγ, TNFα, IL-2, and IL-17A and other pro-inflammatory cytokines were measured in children with confirmed TB (n=36) and unlikely TB (n=46). As shown in **Figure 4A**, the plasma levels of Type 1 and Type 17 cytokines—IFNγ, TNFα, IL-2, and IL-17A were significantly higher in confirmed TB disease compared to unlikely TB children. Other pro-inflammatory cytokines such as IL-6, GM-CSF, IL-1α, and IL-1β showed no statistical differences between the two groups (**Figure 4**).

### Plasma Cytokines Can Robustly Distinguish Confirmed TB Disease From Unlikely TB in the Validation Cohort

To more robustly investigate the discriminatory potential of these candidate immune biomarkers, we next performed ROC analysis of IFNγ, TNFα, IL-2, and IL-17A in confirmed TB vs. unlikely TB children in the validation cohort. As shown in **Figure 4B**, IFNγ, TNFα, IL-2, and IL-17A exhibited significant discriminatory power with high AUC values and sensitivity and specificity in discriminating confirmed TB from unlikely TB children. In comparison to the discovery cohort, the cytokines, TNFα, and IL-17A alone showed good discrimination in confirmed TB compared to unlikely TB.

### Plasma Pro-Inflammatory Cytokine Levels Are Significantly Diminished Following ATT

**Figure 5:**
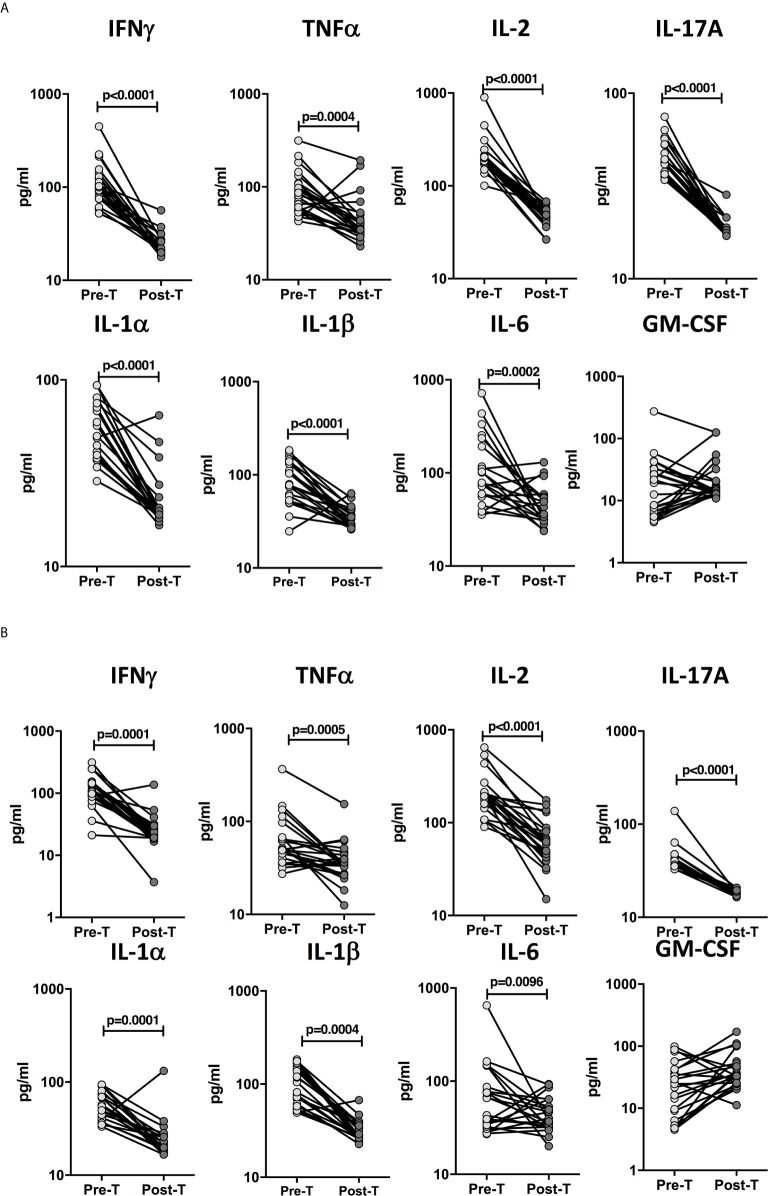
Diminished plasma levels of Type 1, Type 17, and proinflammatory cytokines at the end of standard anti-tuberculosis (TB) therapy in children with confirmed and unconfirmed TB disease. The plasma levels of type 1, type 17, and other proinflammatory cytokines at baseline (pre-T) and at 6 months of anti-TB treatment (post-T) in a subset of **(A)** Confirmed TB (n=24) and **(B)** Unconfirmed TB (n=23) children. The data are presented as line graphs with each line representing a single individual.

To determine whether the elevated levels of pro-inflammatory cytokines are directly associated with TB disease, we determined the levels of these cytokines in a subset of children with confirmed TB (n=24) and unconfirmed TB (n=23) before and after a standard course of ATT (Pre-Treatment (pre-T) versus Post-Treatment (post-T). As shown in **Figure 5A**, in children with confirmed TB at the end of ATT, the plasma levels of IFNγ, TNFα, IL-2, IL-17A, IL-1α, IL-1β, and IL-6 were significantly lower compared to pre-treatment levels. Similarly as shown in **Figure 5B**, in children with unconfirmed TB at the end of ATT, the plasma levels of IFNγ, TNFα, IL-2, IL-17A, IL-1α, and IL-1β were also significantly lower compared to pre-treatment levels.

### A Plasma Signature of Two or Three Cytokines Is an Accurate Biomarker Discriminating Active TB Disease From Unlikely TB

**Figure 6:**
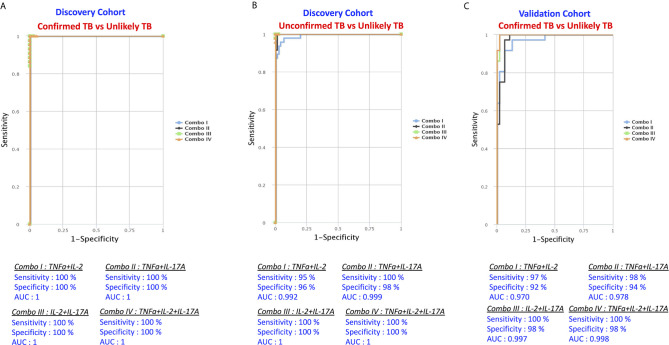
Identification of biomarkers showing the strongest association using combination of cytokine biomarkers in active TB disease. CombiROC model analysis shows the cytokines that exhibited the highest accuracy in discriminating active TB disease from unlikely TB. ROC curves for comparing multiple markers and their combinations between active TB disease versus controls in the Discovery Cohort **(A)** Confirmed TB vs. unlikely TB, **(B)** unconfirmed TB vs. unlikely TB and in the Validation Cohort **(C)** confirmed TB vs. unlikely TB are shown.

The examined cytokines as multiple marker signatures were evaluated in different combinations using CombiROC and the best combinations with the highest AUC, sensitivity and specificity were selected (**Figure 6**). As shown in **Figure 6A**, in the discovery cohort, the dual combination of cytokines TNFα/IL-2 TNFα/IL-17A, IL-2/IL-17A, and triple combination of cytokines TNFα/IL-2/IL-17A showed good significant discriminatory power with high AUC, sensitivity and specificity in discriminating confirmed TB from unlikely TB children. Similarly, as shown **Figure 6B** in, the dual combination of cytokines TNFα/IL-2, TNFα/IL-17A, IL-2/IL-17A, and triple combination of cytokines TNFα/IL-2/IL-17A showed good significant discriminatory power with high AUC, sensitivity and specificity in discriminating unconfirmed TB from unlikely TB children. Finally in the validation cohort as shown in **Figure 6C**, the dual combination of cytokines TNFα/IL-2, TNFα/IL-17A, IL-2/IL-17A, and triple combination of cytokines TNFα/IL-2/IL-17A showed good significant discriminatory power with high AUC, sensitivity and specificity in discriminating confirmed TB from unlikely TB children. Overall, we found that a biomarker signature of two or three cytokines exhibited excellent predictive performance in both discovery and validation cohort.

### Discussion

The immune response associated with distinct clinical forms of TB has been broadly described for the adult population (8). However, studies describing immune determinants of active TB disease in children are uncommon (19). The examination and development of new TB diagnostics that are appropriate for children has been underlined as an important research priority for the End TB Strategy by the WHO (20) and also there is need for point-of-care non-sputum-based test capable of detecting all forms of TB by identifying characteristic biomarkers (21). Few studies have evaluated the diagnostic performance of the correlate of risk (COR) for identifying prevalent TB disease and the prognostic performance of the COR for predicting incident TB disease (22). Development of an improved diagnostic tool for pediatric TB still remains a great challenge, especially since it is a population with immature immune system as well as of high risk of mortality associated with TB (23). We sought to fill this knowledge gap by examining plasma cytokines in children with microbiology confirmed TB, unconfirmed TB and unlikely TB children.

Our study adds to the existing knowledge in the field by demonstrating that plasma immune biomarkers like IFNγ, TNFα, IL-2, and IL-17A can reliably distinguish confirmed TB or unconfirmed TB from unlikely TB control children in a highly endemic region. Overall, results from both the discovery and validation cohort demonstrated that Type 1 cytokines, IFNγ, TNFα, and IL-2 were significantly elevated in confirmed and unconfirmed TB in comparison to those without TB disease but with other respiratory ailments. While it is clear that Th1 cytokines are important in the cell-mediated response to *M.tb* (24), it is also obvious that this particular immune response alone is not sufficient. Previous studies, including our own have reported results regarding the diagnostic potential of Type 1 cytokine biomarkers to distinguish children with TB infection from those who are uninfected in a population with endemic TB (15, 25). Other published studies in HIV-TB coinfection have also reported that IL-2 was an important biomarker of prevalent tuberculosis in HIV infected persons (26). This is further confirmed by the fact that Type 1 cytokine levels are significantly diminished after successful completion of anti-TB treatment. Further, in order to test the diagnostic efficacy of these markers, ROC curve was applied in both the clinical cohorts. The results show that in both the clinical cohorts when comparing confirmed with unlikely TB, TNFα very clearly discriminated the active TB children from unlikely TB controls with more than 90% sensitivity, specificity and AUC of 0.98, whereas IFNγ and IL-2 showed a good sensitivity, specificity and AUC only in the discovery cohort but not in the validation cohort. To overcome this we used combiROC model by utilizing more than one cytokine for better sensitivity and specificity. The results of this study further confirm that our diagnostic Type 1 cytokine biomarkers perform well in this population with a greater background prevalence of LTB at the population level.

Interestingly, in both the discovery and validation cohort, we found significantly elevated levels of IL-17A in confirmed or unconfirmed TB vs unlikely TB controls at homeostasis. We have previously reported that children with TB have diminished IL-17A at baseline or upon TB antigen stimulation compared to controls in culture supernatants (27). In this study, we report that IL-17A was significantly increased in children with confirmed or unconfirmed TB compared to controls, this is in agreement with recent reports of enhanced IL-17A production in adults with active TB (16). Next, in order to assess the diagnostic performance of Type 17 cytokines, ROC curve was applied in both the clinical cohorts when comparing confirmed or unconfirmed TB with unlikely TB. IL-17A was shown to very clearly discriminate active TB children from unlikely TB controls with 100% sensitivity, more than 90% specificity and AUC of 0.98 in both discovery and validation cohort. To our knowledge, this is the only pediatric TB immune biomarker study which showed high sensitivity and specificity to distinguish confirmed TB from unlikely TB children. In addition, we also speculate that higher expression of IL-17A reflects heightened inflammation in these children, which denotes higher than the optimal expression which may not play a protective role. When measuring other pro-inflammatory cytokines, we found significantly elevated levels of IL-1α, and IL-6 in confirmed TB or unconfirmed TB vs unlikely TB at baseline in the discovery cohort but not in the validation cohort. Even upon stratification by age, we continued to observe significant changes between the confirmed TB and unlikely TB children.

Next, we utilized a combination of biomarkers in evaluating the combined performance of all the tested immune signatures. We created all possible cytokine combinations for each active TB group with respect to controls in the both discovery and validation cohort. The combinations that delivered the highest sensitivity and specificity values were considered for selection of efficient immune biomarker signatures. The multi-biomarker panel of TNFα/IL-2/IL-17A or IL-2/IL-17A offered the highest values for sensitivity and specificity and AUC in both the clinical cohorts. Our study suffers from certain limitations, including the use of pediatric TB children from the same geographic region (albeit from two different hospitals), limited sample size due to our use of only microbiologically confirmed TB as the gold standard and lack of blood collection during treatment follow up in the validation cohort and measurement of two to three cytokines in plasma would appear to present an economic barrier.

Nevertheless, this study benefits by the use of two discrete clinical cohorts of children. The strengths of this study involve the use of well-characterized participant groups and, critically, the inclusion of an unlikely TB group enabling us to determine the high sensitivity and specificity of the cytokine responses between the confirmed TB/unconfirmed TB vs unlikely TB. In addition, our study also innovates by using a group of children with only unconfirmed (clinically/radiologically confirmed TB) and demonstrating that the biomarker panels are equally adept at diagnosing this group. This provides an important tool to tackle the menace of pediatric TB since a major part of diagnosing and treating TB is done empirically at the discretion of the clinician without microbiological confirmation. Our plasma cytokine biosignature, therefore would be a great value addition to the field of pediatric TB diagnosis and treatment. In conclusion, our current results report that children in a highly TB endemic setting, three cytokine biomarkers (IL-2, TNF-α, and IL-17A) using Combi ROC model or single cytokines had the ability to discriminate between active TB disease and non-TB disease in children with high levels of sensitivity and specificity. Finally, to our knowledge, this is the first study to show that plasma biomarkers can serve as a diagnostic test meeting the WHO specified target product profile (100% sensitivity and 98–100% specificity in this case) (28). Further successful validation of these immune biomarkers in different pediatric populations and different geographical regions could serve as the basis to develop a rapid POC test for this menacing disease.

## Data Availability Statement

The original contributions presented in the study are included in the article/**Supplementary Material**. Further inquiries can be directed to the corresponding authors.

## Ethics Statement

The studies involving human participants were reviewed and approved by NIRT Institutional Ethics Committee. Written informed consent to participate in this study was provided by the participants’ legal guardian/next of kin.

## Author Contributions

Designed the study (SB, NK, SH). Conducted experiments (NK, GS). Acquired data (NK, KT). Analyzed data (NK, KT). Contributed reagents and also revised subsequent drafts of the manuscript (SH, SS, ST, SB). Responsible for the enrolment of participant and also contributed to acquisition and interpretation of clinical data (VB, NS, JS, ES, KS, GJ, AA, DB). Wrote the manuscript (SB, NK). All authors contributed to the article and approved the submitted version.

## Funding

This work was supported by the Division of Intramural Research, National Institute of Allergy and Infectious Diseases (NIAID). And this study was also partially supported by USAID, WHO and ICMR under Model DOTS project. The funders had no role in study design, data collection and analysis, decision to publish, or preparation of the manuscript.

## Conflict of Interest

The authors declare that the research was conducted in the absence of any commercial or financial relationships that could be construed as a potential conflict of interest.
