# Supplementary material for: Discovery and Validation of a Three-Cytokine Plasma Signature as a Biomarker for Diagnosis of Pediatric Tuberculosis
Source: Front Immunol. 2021 Apr 16;12:653898. doi: 10.3389/fimmu.2021.653898 (PMC8085486; doi:10.3389/fimmu.2021.653898)
Supplement: Supplementary file 1 [file DataSheet_1.pdf]

Table S1

PID	Alternative Diagnosis	Diagnosis
Control 1	Bacterial pneumonia	Symptoms+X-ray not suggestive of TB
Control 2	Viral pneumonia	Symptoms+X-ray not suggestive of TB
Control 3	Viral pneumonia	Symptoms+X-ray not suggestive of TB
Control 4	Asthma/wheeze	Symptoms+X-ray not suggestive of TB
Control 5	Asthma/wheeze	Symptoms+X-ray not suggestive of TB
Control 6	Asthma/wheeze	Symptoms+X-ray not suggestive of TB
Control 7	Bacterial pneumonia	Symptoms+X-ray not suggestive of TB
Control 8	Bacterial pneumonia	Symptoms+X-ray not suggestive of TB
Control 9	Viral pneumonia	Symptoms+X-ray not suggestive of TB
Control 10	Viral pneumonia	Symptoms+X-ray not suggestive of TB
Control 11	Viral pneumonia	Symptoms+X-ray not suggestive of TB
Control 12	Viral pneumonia	Symptoms+X-ray not suggestive of TB
Control 13	Asthma/wheeze	Symptoms+X-ray not suggestive of TB
Control 14	Viral pneumonia	Symptoms+X-ray not suggestive of TB
Control 15	Asthma/wheeze	Symptoms+X-ray not suggestive of TB
Control 16	Viral pneumonia	Symptoms+X-ray not suggestive of TB
Control 17	Asthma/wheeze	Symptoms+X-ray not suggestive of TB
Control 18	Asthma/wheeze	Symptoms+X-ray not suggestive of TB
Control 19	Bacterial pneumonia	Symptoms+X-ray not suggestive of TB
Control 20	Asthma/wheeze	Symptoms+X-ray not suggestive of TB
Control 21	Asthma/wheeze	Symptoms+X-ray not suggestive of TB
Control 22	Asthma/wheeze	Symptoms+X-ray not suggestive of TB
Control 23	Viral pneumonia	Symptoms+X-ray not suggestive of TB
Control 24	Viral pneumonia	Symptoms+X-ray not suggestive of TB
Control 25	Asthma/wheeze	Symptoms+X-ray not suggestive of TB
Control 26	Bacterial pneumonia	Symptoms+X-ray not suggestive of TB
Control 27	Bacterial pneumonia	Symptoms+X-ray not suggestive of TB
Control 28	Bacterial pneumonia	Symptoms+X-ray not suggestive of TB
Control 29	Asthma/wheeze	Symptoms+X-ray not suggestive of TB
Control 30	Asthma/wheeze	Symptoms+X-ray not suggestive of TB
Control 31	Viral pneumonia	Symptoms+X-ray not suggestive of TB
Control 32	Viral pneumonia	Symptoms+X-ray not suggestive of TB
Control 33	Viral pneumonia	Symptoms+X-ray not suggestive of TB
Control 34	COPD	Symptoms+X-ray not suggestive of TB
Control 35	COPD	Symptoms+X-ray not suggestive of TB
Control 36	Bacterial pneumonia	Symptoms+X-ray not suggestive of TB
Control 37	Bacterial pneumonia	Symptoms+X-ray not suggestive of TB
Control 38	Bacterial pneumonia	Symptoms+X-ray not suggestive of TB
Control 39	Bacterial pneumonia	Symptoms+X-ray not suggestive of TB
Control 40	Asthma/wheeze	Symptoms+X-ray not suggestive of TB

Control 41	Asthma/wheeze	Symptoms+X-ray not suggestive of TB
Control 42	Viral pneumonia	Symptoms+X-ray not suggestive of TB
Control 43	Viral pneumonia	Symptoms+X-ray not suggestive of TB
Control 44	Viral pneumonia	Symptoms+X-ray not suggestive of TB
Control 45	Viral pneumonia	Symptoms+X-ray not suggestive of TB
Control 46	COPD	Symptoms+X-ray not suggestive of TB
Control 47	Bacterial pneumonia	Symptoms+X-ray not suggestive of TB
Control 48	Bacterial pneumonia	Symptoms+X-ray not suggestive of TB
Control 49	Bacterial pneumonia	Symptoms+X-ray not suggestive of TB
Control 50	Bacterial pneumonia	Symptoms+X-ray not suggestive of TB
Control 51	Asthma/wheeze	Symptoms+X-ray not suggestive of TB
Control 52	Asthma/wheeze	Symptoms+X-ray not suggestive of TB
Control 53	Asthma/wheeze	Symptoms+X-ray not suggestive of TB
Control 54	Viral pneumonia	Symptoms+X-ray not suggestive of TB
Control 55	Viral pneumonia	Symptoms+X-ray not suggestive of TB
Control 56	Viral pneumonia	Symptoms+X-ray not suggestive of TB
Control 57	Viral pneumonia	Symptoms+X-ray not suggestive of TB
Control 58	Asthma/wheeze	Symptoms+X-ray not suggestive of TB
Control 59	Asthma/wheeze	Symptoms+X-ray not suggestive of TB
Control 60	Asthma/wheeze	Symptoms+X-ray not suggestive of TB
Control 61	Asthma/wheeze	Symptoms+X-ray not suggestive of TB
Control 62	Bacterial pneumonia	Symptoms+X-ray not suggestive of TB
Control 63	Bacterial pneumonia	Symptoms+X-ray not suggestive of TB
Control 64	Bacterial pneumonia	Symptoms+X-ray not suggestive of TB
Control 65	Bacterial pneumonia	Symptoms+X-ray not suggestive of TB
Control 66	Viral pneumonia	Symptoms+X-ray not suggestive of TB
Control 67	Asthma/wheeze	Symptoms+X-ray not suggestive of TB
Control 68	Asthma/wheeze	Symptoms+X-ray not suggestive of TB
Control 69	Viral pneumonia	Symptoms+X-ray not suggestive of TB
Control 70	Viral pneumonia	Symptoms+X-ray not suggestive of TB
Control 71	Viral pneumonia	Symptoms+X-ray not suggestive of TB
Control 72	Viral pneumonia	Symptoms+X-ray not suggestive of TB
Control 73	Viral pneumonia	Symptoms+X-ray not suggestive of TB
Control 74	Viral pneumonia	Symptoms+X-ray not suggestive of TB
Control 75	Viral pneumonia	Symptoms+X-ray not suggestive of TB
Control 76	Asthma/wheeze	Symptoms+X-ray not suggestive of TB
Control 77	Asthma/wheeze	Symptoms+X-ray not suggestive of TB
Control 78	Asthma/wheeze	Symptoms+X-ray not suggestive of TB
Control 79	Viral pneumonia	Symptoms+X-ray not suggestive of TB
Control 80	Bacterial pneumonia	Symptoms+X-ray not suggestive of TB
Control 81	Bacterial pneumonia	Symptoms+X-ray not suggestive of TB
Control 82	Bacterial pneumonia	Symptoms+X-ray not suggestive of TB
Control 83	Bacterial pneumonia	Symptoms+X-ray not suggestive of TB
Control 84	Viral pneumonia	Symptoms+X-ray not suggestive of TB
Control 85	Viral pneumonia	Symptoms+X-ray not suggestive of TB

Control 86	Viral pneumonia	Symptoms+X-ray not suggestive of TB
Control 87	Bacterial pneumonia	Symptoms+X-ray not suggestive of TB
Control 88	Bacterial pneumonia	Symptoms+X-ray not suggestive of TB
Control 89	Viral pneumonia	Symptoms+X-ray not suggestive of TB
Control 90	Asthma/wheeze	Symptoms+X-ray not suggestive of TB
Control 91	Asthma/wheeze	Symptoms+X-ray not suggestive of TB
Control 92	Asthma/wheeze	Symptoms+X-ray not suggestive of TB
Control 93	Bacterial pneumonia	Symptoms+X-ray not suggestive of TB
Control 94	Bacterial pneumonia	Symptoms+X-ray not suggestive of TB
Control 95	Bacterial pneumonia	Symptoms+X-ray not suggestive of TB
Control 96	Viral pneumonia	Symptoms+X-ray not suggestive of TB
Control 97	Bacterial pneumonia	Symptoms+X-ray not suggestive of TB
Control 98	Viral pneumonia	Symptoms+X-ray not suggestive of TB
Control 99	Viral pneumonia	Symptoms+X-ray not suggestive of TB
Control 100	Viral pneumonia	Symptoms+X-ray not suggestive of TB
Control 101	Viral pneumonia	Symptoms+X-ray not suggestive of TB
Control 102	Viral pneumonia	Symptoms+X-ray not suggestive of TB
Control 103	Bacterial pneumonia	Symptoms+X-ray not suggestive of TB
Control 104	Bacterial pneumonia	Symptoms+X-ray not suggestive of TB
Control 105	Bacterial pneumonia	Symptoms+X-ray not suggestive of TB
Control 106	Bacterial pneumonia	Symptoms+X-ray not suggestive of TB
Control 107	Bacterial pneumonia	Symptoms+X-ray not suggestive of TB
Control 108	Bacterial pneumonia	Symptoms+X-ray not suggestive of TB
Control 109	Bacterial pneumonia	Symptoms+X-ray not suggestive of TB
Control 110	Viral pneumonia	Symptoms+X-ray not suggestive of TB
Control 111	Viral pneumonia	Symptoms+X-ray not suggestive of TB
Control 112	Viral pneumonia	Symptoms+X-ray not suggestive of TB
Control 113	Viral pneumonia	Symptoms+X-ray not suggestive of TB
Control 114	Viral pneumonia	Symptoms+X-ray not suggestive of TB
Control 115	Asthma/wheeze	Symptoms+X-ray not suggestive of TB
Control 116	Asthma/wheeze	Symptoms+X-ray not suggestive of TB
Control 117	Bacterial pneumonia	Symptoms+X-ray not suggestive of TB
Control 118	Asthma/wheeze	Symptoms+X-ray not suggestive of TB
Control 119	Viral pneumonia	Symptoms+X-ray not suggestive of TB
Control 120	Asthma/wheeze	Symptoms+X-ray not suggestive of TB
Control 121	Bacterial pneumonia	Symptoms+X-ray not suggestive of TB
Control 122	Bacterial pneumonia	Symptoms+X-ray not suggestive of TB

Supplementary table 1: Clinical characteristics of unlikely TB children

Table S2 A

Children <5Years		
**Cytokines**	**Confirmed TB vs Unlikely TB**	**Unconfirmed TB vs Unlikely TB**
**IFNγ**	**p<0.0001**	**p<0.0001**
**IL-2**	**p<0.0001**	**p<0.0001**
**TNFα**	**p=0.0141**	**p<0.0001**
**IL-1α**	**p<0.0001**	**p<0.0001**
**IL-1β**	p>0.9999	p>0.9999
**IL-17A**	**p=0.0074**	**p<0.0001**
**IL-6**	**p=0.023**	**p=0.0013**
**IL-10**	**p<0.0001**	p=0.0316
**GM-CSF**	p>0.9999	p=0.2203
**IL-4**	p=0.4498	p=0.8626
**IL-5**	p>0.9999	p=>0.9999
**IL-13**	p=0.1433	p=>0.9999

Table S2 B

Children >5Years		
**Cytokines**	**Confirmed TB vs Unlikely TB**	**Unconfirmed TB vs Unlikely TB**
**IFNγ**	**p<0.0001**	**p<0.0001**
**IL-2**	**p<0.0001**	**p<0.0001**
**TNFα**	**p=0.0006**	**p<0.0001**
**IL-1α**	**p=0.0004**	**p<0.0001**
**IL-1β**	p>0.9999	**p=0.0057**
**IL-17A**	**p<0.0001**	**p<0.0001**
**IL-6**	**p=0.0001**	**p<0.0001**
**IL-10**	**p=0.0001**	**p<0.0001**
**GM-CSF**	p=0.0862	**p=0.0047**
**IL-4**	p>0.9999	**p=0.0015**
**IL-5**	p=0.0608	p=0.1353
**IL-13**	p=0.0538	p=0.0948

Supplementary table 2: The plasma levels of IFNγ, TNFα, IL-2, IL-17A, IL-1α, IL-1β, GM-CSF and IL-6 were measured in confirmed TB (n=80) and unlikely TB (n=122) children from both the clinical cohorts together. (A) Children less than 5-year-old (<5 Years) and (B) children greater than 5 year old (>5 Years) at baseline. P values were calculated using the Mann-Whitney test with Holm's correction for multiple comparisons.
